# Coherently remapping toroidal cells but not Grid cells are responsible for path integration in virtual agents

**DOI:** 10.1016/j.isci.2023.108102

**Published:** 2023-09-30

**Authors:** Vemund Schøyen, Markus Borud Pettersen, Konstantin Holzhausen, Marianne Fyhn, Anders Malthe-Sørenssen, Mikkel Elle Lepperød

**Affiliations:** 1 Department of Physics, University of Oslo, Oslo 0313, Norway; 2 Department of Biosciences, University of Oslo, Oslo 0313, Norway; 3 Simula Research Laboratory, Norway

**Keywords:** Machine learning, Neuroscience, Systems biology

## Abstract

It is widely believed that grid cells provide cues for path integration, with place cells encoding an animal’s location and environmental identity. When entering a new environment, these cells remap concurrently, sparking debates about their causal relationship. Using a continuous attractor recurrent neural network, we study spatial cell dynamics in multiple environments. We investigate grid cell remapping as a function of global remapping in place-like units through random resampling of place cell centers. Dimensionality reduction techniques reveal that a subset of cells manifest a persistent torus across environments. Unexpectedly, these toroidal cells resemble band-like cells rather than high grid score units. Subsequent pruning studies reveal that toroidal cells are crucial for path integration while grid cells are not. As we extend the model to operate across many environments, we delineate its generalization boundaries, revealing challenges with modeling many environments in current models.

## Highlights

• RNNs trained for path integration in three environments learned a persistent torus
• Ablating toroidal cells disrupted the torus structure and impaired path integration
• Ablating grid cells had much lower impact on path integration performance compared to toroidal cells
• Toroidal cells coherently phase remaped when place cells remapped globally

## Graphical abstract

**Figure:**
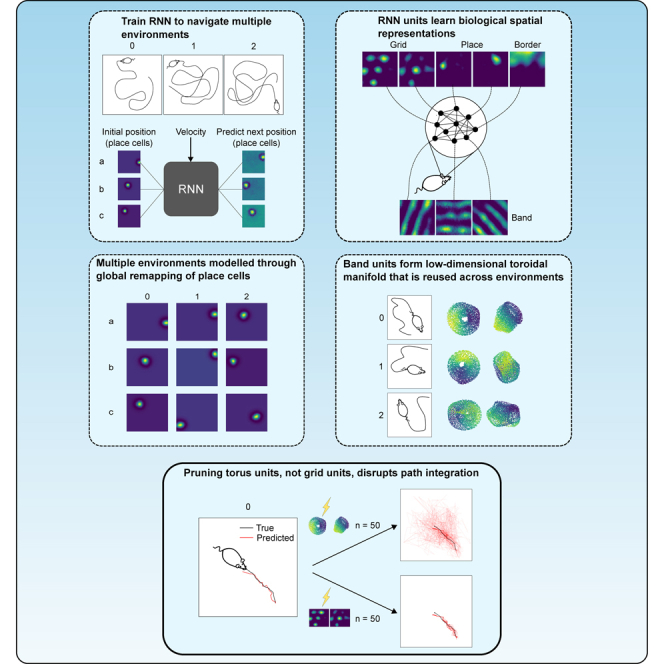

## Introduction

Navigation is a complex problem that involves multiple sensory modalities and subtasks. One such subtask is path integration, relying on self-motion cues, which is thought to be supported by grid cells.^1^^,^^2^^,^^3^^,^^4^^,^^5^^,^^6^^,^^7^^,^^8^ Grid cells are arranged in subpopulations, called modules.^9^ The collective activity of each module has been both theoretically,^3^^,^^10^ and experimentally,^11^ shown to form a low dimensional, twisted torus manifold. Another feature of grid cells is their persistent activity across environments. While activity is persistent, grid cells can exhibit changes in their firing characteristics through coherent shifts in phase and orientation when an animal is moved from one environment to another.^12^ Together, this suggests that a population of grid cells may constitute a path integration system that generalizes across environments.

Apart from general path integration mechanisms, navigating multiple environments requires distinguishing between them. In the brain, place cells of the hippocampus are thought to encode not only the allocentric location of an animal, but also the identity of the environment.^13^ Collectively, the activity of an ensemble of place cells is believed to encode a cognitive map of the environment.^14^ The process of switching between such maps is referred to as remapping. Unlike grid cells, the population activity of hippocampal place cells displays non-coherent variability between environments. For example, place cells may alter their preferred firing locations and rates when moved between different recording enclosures.^12^^,^^13^^,^^15^^,^^16^

Place and grid cells display distinct remapping behaviours, but have been shown to remap concurrently.^12^ This has led to an ongoing debate concerning the causal relationship between the two cell types. One popular theory holds that place field formation and remapping are primarily driven by upstream grid cell activity and remapping.^17^^,^^18^^,^^19^ This is supported by the presence of strong projections from layers II and III of the Medial Entorhinal Cortex (MEC) to the dorsal hippocampus. However, the grid-to-place model of place field formation line of argumentation has been called into question.^20^^,^^21^ First, there is experimental evidence that place cells form before grid cells in development.^22^^,^^23^^,^^24^ Second, place cell remapping can occur without MEC input.^25^ Third, reciprocal connections exist between the hippocampus and the MEC.^20^^,^^26^ Finally, silencing the dorsal hippocampus has detrimental effects on grid cells.^27^ To further resolve this debate it is necessary to evaluate if place cell remapping is sufficient to explain grid cell remapping, a problem well suited for computational study.

There has been a recent surge in the development of computational models of the hippocampal area.^28^^,^^29^^,^^30^^,^^31^^,^^32^^,^^33^^,^^34^^,^^35^ However, the connection between place and grid cell remapping remains unresolved. It has been shown that hand-tuned continuous attractor neural network (CANN) models reproduce the spatial firing pattern of grid cells, while performing accurate path integration.^3^^,^^10^ However, these models typically do not model the interplay between place and grid cells. More recently, normative recurrent neural network models trained to path integrate have been shown to learn emergent grid-like spatial representations.^28^^,^^29^^,^^30^^,^^35^ Moreover, Sorscher et al. showed that such a model learned a representation resembling a continuous attractor, henceforth referred to as a continuous attractor recurrent neural network (CARNN). Importantly, the CARNN model accommodates both place and grid-like units, providing a suitable foundation for studying the remapping problem.

In this study, we extended the model proposed by Sorscher et al. to navigate multiple environments due to its flexibility and the ability to model the interplay between place and grid cells. Our approach randomly redistributed the locations of place cell-like units, similar to place cell global remapping. This allowed us to study grid cell remapping as a consequence of place remapping in a multi-environment setting and enabled us to explicitly test whether grid cell remapping can be explained as the result of global remapping in hippocampal cells.

To explore how the network manages to path integrate in multiple environments, we leveraged recent dimensionality reduction techniques.^11^^,^^36^ We found that the activity of a subpopulation of units in the trained network formed a low-dimensional toroidal manifold that persisted across environments. We also found that the learned cell types that collectively set up the torus resemble band-like cells and contained few cells with high grid scores. To investigate the importance of different cell types for path integration, we employed pruning strategies. Finally, we further extended the model to many environments (up to 50) to explore the limits of the network’s ability to generalise.

## Results

### Model and dataset

**Figure 1:**
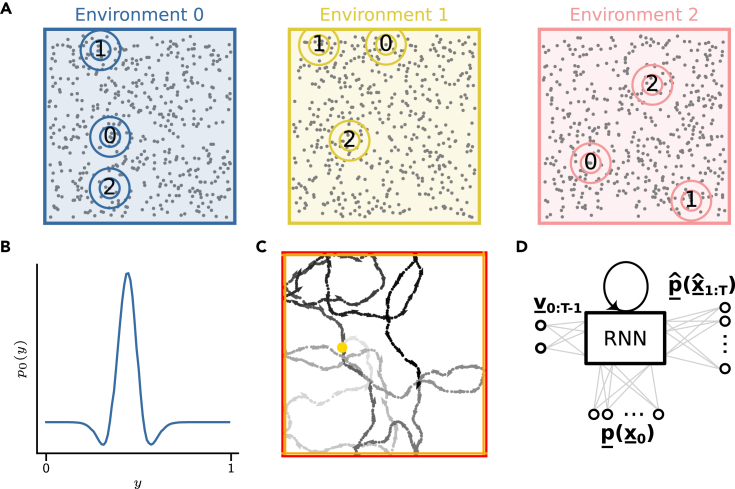
The Continuous Attractor Recurrent Neural Network (CARNN) and global remapping (A) Three environments with identical geometry but different, randomly distributed place cell centers. Circles with numbers indicate the identity of a cell, and the figure shows how each cell is remapped to other positions in the other environments. (B) Spatial profile of a single place cell firing field, isolated from an ensemble. The profile is a 1D cross-section of a 2D Mexican hat. (C) An example of a random path within an environment. The initial position is given by the yellow dot. The red and orange lines define the hard and soft boundaries of the environment, respectively. Arrows along the trajectory indicate the agent’s current head direction and speed. Color intensity indicates time, with lighter greys corresponding to earlier steps in the trajectory. (D) Schematic of the model architecture following Sorscher et al. The initial position of the trajectory ${\underline{x}}_{0}$ in place cell representation $\underline{p}\left({\underline{x}}_{0}\right)$ is linearly fed into the recurrent layer, which sets the initial state of the RNN. The network updates its internal state using a sequence of Cartesian velocity vectors ${\left\{{\underline{v}}_{t}\right\}}_{t\in \left\{1,2,\ldots ,T\right\}}$ and predicts subsequent positions in a place cell representation $\hat{\underline{p}}\left({\hat{\underline{x}}}_{T}\right)$, through path integration.

To simulate navigation across multiple environments we extended the model introduced by Sorscher et al.,^30^ as depicted in (Figure 1D), (see The model for a detailed model description). In this model, navigation amounts to path integration based on self-motion signals. Path integration was achieved by training the network to self-localize along simulated trajectories. To generate trajectories inspired by the foraging behaviour of rodents we also developed a trajectory simulation tool (rat-simulator, see generating dataset trajectories, and Figure 1C) for an example trajectory). Using this toolkit, we were able to generate multiple trajectories in multiple environments by associating trajectories with the activity of distinct place cell ensembles.

We modeled multi-environment navigation using global place cell remapping by rearranging both the input and label distribution firing locations according to a random uniform distribution (Figure 1A). While firing locations were randomly redistributed, the functional form of the place cell firing profile (Figure 1B) was maintained across environments, emulating global remapping of place cells. Accordingly, an environment was uniquely characterized by the place cell ensemble’s center locations and the associated activity. The architecture of the model, however, remained the same as proposed by Sorscher et al. Allowing the initial input and label distributions to remap between environments, we can study whether grid cell remapping emerges as a consequence of place cell remapping.

### Grid cells emerge during training for path integration in multiple environments

**Figure 2:**
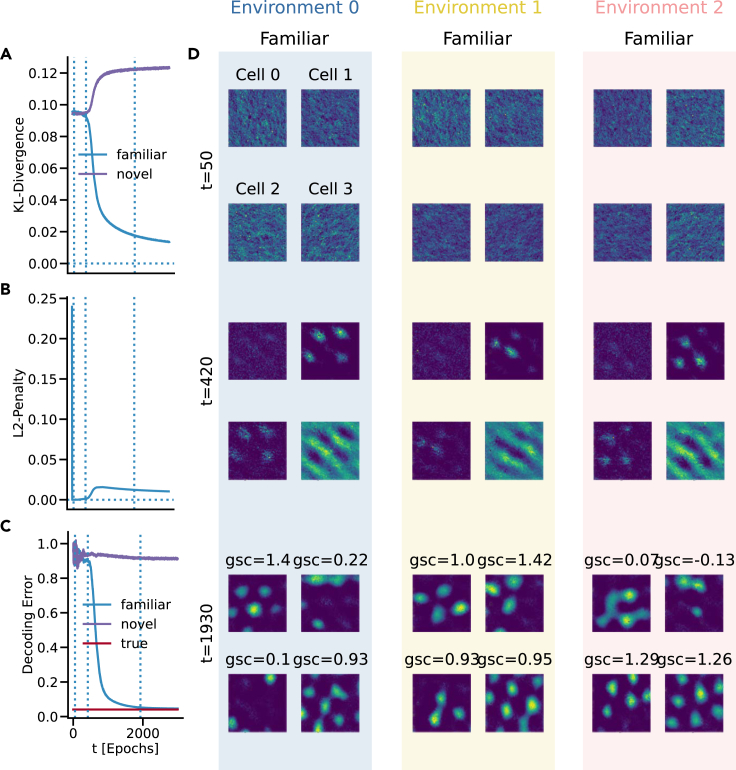
Training metrics and firing fields of selected cells in the recurrent layer of the model (A) KL divergence versus training time *t* (in epochs) measuring the discrepancy between predicted and place cell population activity. (B) L2 penalty of recurrent weights during training. (C) Distance between predicted and estimated true position in decoded Euclidean coordinates during training. (D) Firing fields at three stages of training for three units with high GCS across environments, as well as one randomly selected unit.

When trained in three distinct environments, the network learned the place cell label distributions, as evidenced by the KL divergence of the familiar environments in Figure 2A. Similarly, the position decoding error approached the baseline decoding error (Figure 2C), indicating that the network learned to path integrate in all three environments. In the novel environment, where the model encountered a fourth, unfamiliar resampling of the place cell center locations, path integration performance remained close to its initial value. The L2-penalty curve (Figure 2B) shows how the magnitude of recurrent connections evolves during training.

When assessing the recurrent units of the network we found that they learned spatially selective responses in all three environments, including hexagonal, grid-like responses. Figure 2D shows example tuning profiles for trained units with high grid scores in all environments. Moreover, the training dynamics display three distinct phases: (1) The first training phase (t=50) is characterized by a rapid decrease in the L2 penalty (Figure 2B) and small changes in the KL-divergence; the corresponding cell ratemaps also display little spatial tuning (Figure 2D, top row). (2) The second phase of training (t=420) features an increase in the L2-penalty and a concurrent decrease in KL-divergence. Notably, the units of the recurrent network begin to exhibit signs of spatial selectivity at this stage of training (Figure 2D, middle row). (3) The final phase of training (t=1930) indicates training convergence, both in terms of KL divergence and L2 penalty. Figure 2D demonstrates that units now display strong spatial selectivity. In particular, some trained units display hexagonal, grid-like firing patterns (e.g., top left unit in environment 0, bottom row; grid score = 1.4).

We proceeded to assess the recurrent unit activities across environments. Notably, the same unit can exhibit distinct firing fields in different environments. Units can display both heterogeneous and grid-like spatial responses (e.g., bottom row, top left unit), or can appear consistently grid-like, but exhibit shifted firing fields (bottom right unit) when compared across environments. See Figures S7–S9 for more examples of unit ratemaps in the three environments.

These findings show that units are reused across environments, and that some maintain their functional form. Collectively, these results indicate that the network learns diverse unit types, and that the responses of these units may remap to solve the navigation task in multiple environments.

### Toroidal cells are crucial for path integration

Following the same units across environments we observed consistent functional forms which motivated us to assess the corresponding population activity. Through the clustering method outlined in clustering and grid module identification, we identified 18 distinct cell clusters (which can be seen in Figure S1). Using dimensionality reduction techniques detailed in low dimensional projection of cell clusters, we identified three clusters resembling band-like cells that collectively encoded a toroidal structure; we refer to these as ‘toroidal cells’ (see clustered band cells in Figure S5). Out of a total population of 4096, 604 cells were categorized as belonging to this torus ensemble. The intersection between the toroidal and the 604 highest-ranking grid cells comprises 56 cells, which is significantly fewer than expected by pure chance. Using a Binomial model, we calculate the probability of counting less than 56 cells overlapping between two sets of 604 cells out of 4096 to be of order $P\left(X<=56\right)=O\left(10^{-4}\right)$. This comparably low probability suggests that these subpopulations have significantly fewer units in common than expected by chance.

**Figure 3:**
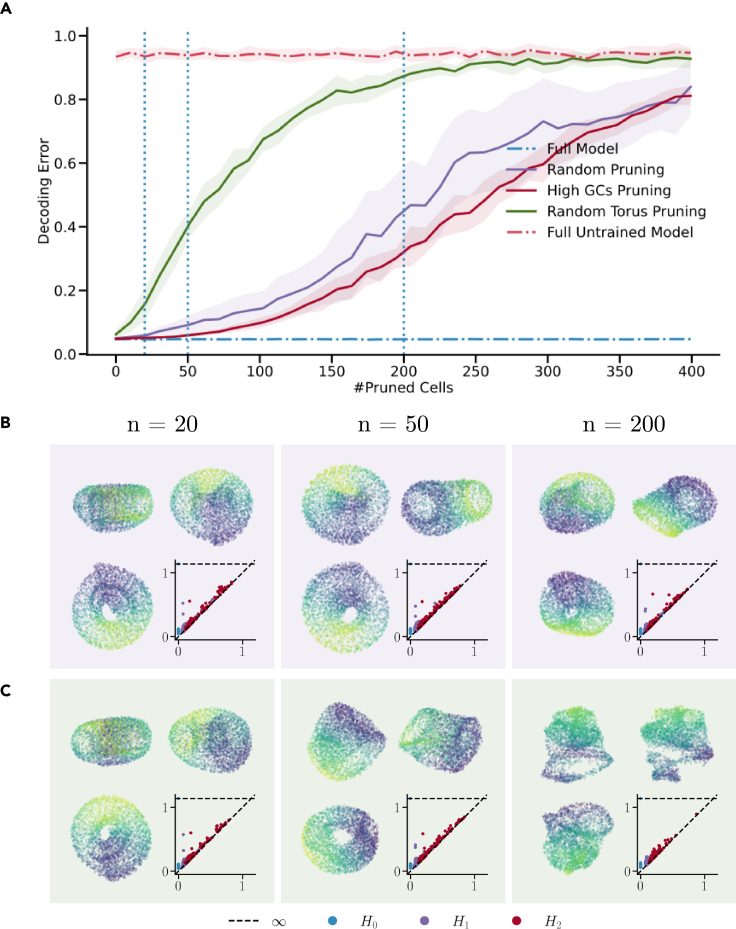
Toroidal cells are crucial for path integration while high grid score or randomly selected cells are less important (A) Decoding error as a function of pruning. Curves and error shadings are given as median ± median absolute deviation. (B) Low-dimensional projection of toroidal cells when pruning 20, 50, and 200 random cells. Each stage shows the manifold at three different viewing angles. The color is given by the value of the first linear principal component (PCA). The persistence diagram quantifies zero, one and two-dimensional co-cycles. (C) Same as (A) but when pruning toroidal cells.

We further assessed these cells’ importance with regard to path integration performance using pruning techniques (see pruning for a detailed description). Figure 3A displays how path integration performance deteriorates as we increase the amount of pruned cells using different strategies. The ‘Full Model’ and ‘Full Untrained Model’ provide the best and worst-case scenarios without pruning, respectively. We observe that pruning either random or high grid units has a similar impact on path integration ability. Notably, pruning toroidal cells increases decoding error at a higher rate (Figure 3C). For completeness, we also provide pruning curves for all clusters, shown in Figure S1. In addition, Figure S2 demonstrates the decoding error as a function of time, for a fixed number of pruned units. Overall, the presented results provide compelling evidence of the crucial role of toroidal cells in path integration.

Ablating toroidal cells highlights their impact on decoding error. However, to understand their relation to the population activity we also assessed the structural integrity of the population-induced toroid under ablation. During random unit pruning (Figure 3B), the structural integrity of the toroidal manifold is preserved at all three pruning levels, as shown by the persistence of specific cocycles. After pruning 50 toroidal cells (Figure 3C), the torus remained, but one of its holes become less persistent. In contrast, after removing 200 toroidal cells, the low-dimensional representation of the remaining cells morphed into a complex shape. This results in both one-dimensional and two-dimensional cocycles losing persistence, signifying the breakdown of the torus. Notably, when the toroidal structure is compromised, there’s an almost total loss of the network’s ability to perform path integration.

### Toroidal cells persist and remap coherently across environments

**Figure 4:**
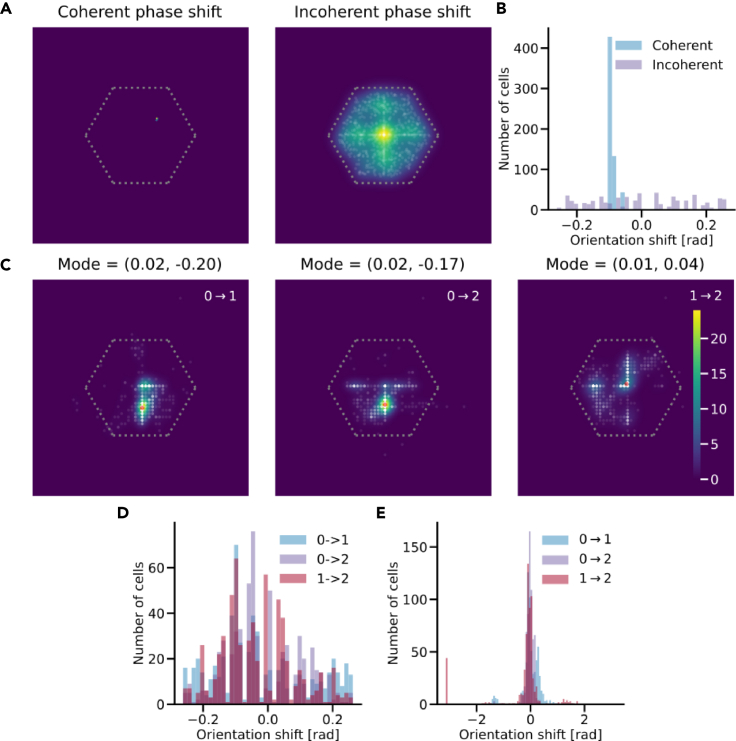
Ideal grid cell and learned toroidal cell remapping (A) Ideal (in)coherent phase-shifts. (B) Ideal (in)coherent orientation shifts in the −15 to 15° range. (C) Toroidal cell phase-shifts. (D) Toroidal cell orientation shifts in the −15 to 15° range. (E) Toroidal cell orientation shifts in the full −180 to 180° range.

As seen in Figure 2, network units change their spatial representations across different environments. This observation led us to investigate whether populations maintained their functional form during environmental change, in a manner consistent with experimentally observed remapping. We, therefore, measured the extent to which toroidal cells undergo shifts in both orientation and phase. For comparison, and to build some intuition, we modeled remapping in ideal grid cell modules; see synthetic grid cells for details. We present examples to distinguish between coherent and incoherent phase shifts in Figure 4A using the method described in grid phase shift. Similarly, examples of orientation shifts are provided in Figure 4B, based on the method detailed in grid orientation shift. Ideal coherent shifts are characterized by pronounced, unimodal peaks. In contrast, incoherent shifts tend to have a nearly uniform distribution.

**Figure 5:**
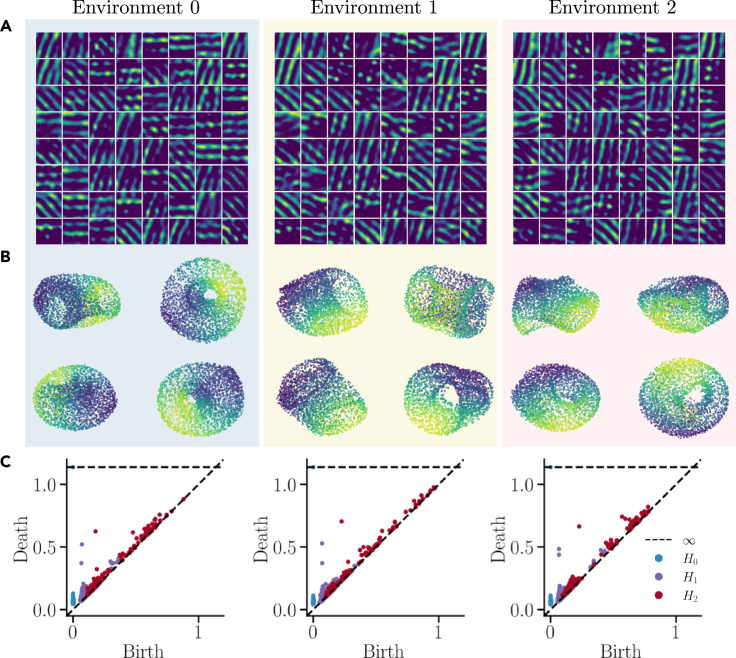
Toroidal cells persistently encode a torus in all three environments (A) Example ratemaps of toroidal cells in the three environments. (B) Low-dimensional projection of the toroidal cells in the three environments. Each environment shows the same point cloud at four viewing angles. (C) Persistence diagrams for the toroidal cells in the three environments.

Figure 4C shows calculated phase shifts for all toroidal cells and environments. For reference, ratemaps of example toroidal cells are showcased in Figure 5A for each environment. Notably, transitions from environment 0 to the other environments predominantly exhibit a coherent phase shift along the cardinal y-axis. The phase shift observed between environments 1 and 2 is somewhat less coherent, yet it accumulates near-zero phase shifts. Overall, the remapping of toroidal cells seems to either undergo coherent phase shifts or maintain stability across different environments.

Figure 4D presents wrapped orientation shifts ranging between −15 and 15° for all toroidal cells. A majority of these shifts amass near 0 (mean values −1.38, −0.84, and −1.84 degrees for transitions between environments 0→1, 0→2, and 1→2 respectively). This clustering suggests a predominant zero-shift in orientation when moving from one environment to another. In Figure 4E, these orientation shifts are detailed across the full spectrum of 360°. In other words, toroidal cells appear to maintain their orientation across environments.

We further confirm the consistency of toroidal cells (Figure 5A) by investigating their ensemble low-dimensional representations in the three environments. By visual inspection (Figure 5B) and quantitative verification (Figure 5C) we observe that they encode a torus in all three environments. For comparison, we performed a similar analysis with high grid score cells (Figure S3A). These cells neither appear to encode a simple low-dimensional geometry, nor consistently retain their shape between environments (Figure S3B) compared to (Figure 4B). However, the high grid score population showed a somewhat coherent phase shift (Figure S3C) and orientation shift (Figure S3D). Combined, these results indicate that the toroidal population seems more comparable to experimental evidence than the high grid score counterpart.

### Many environments and continual learning degrade pattern formation and training metrics

The fact that we observe a torus throughout environments in the multi-learning setting suggests that this structure is universal. If the model was able to reuse this structure, the model could potentially be extended to many environments without a drop in performance. Therefore, we extend the model to learn many (10 and 50) environments and study the effect on training metrics and spatial representations.

**Figure 6:**
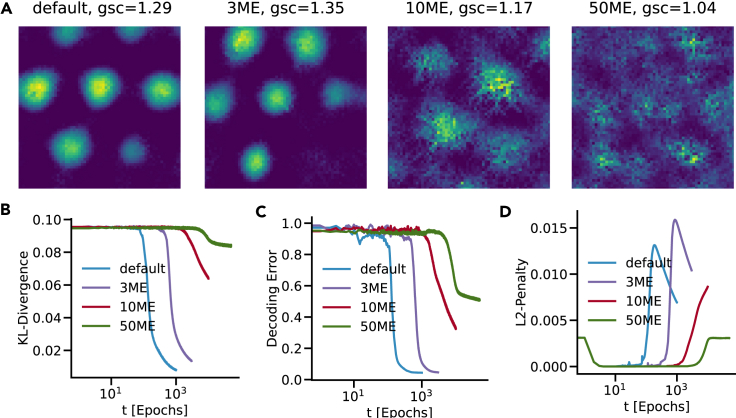
Probing the model’s ability to generalize to many environments (A) Example ratemaps for high grid score units in one (default), three (3ME), ten (10ME) and fifty (50ME) environments. (B–D) (B) KL divergence, (C) decoding error and (D) and L2-penalty training metrics for each model.

Figure 6A shows one example of a high grid score cell in a model that was trained in 1 (default), 3, 10 and 50 environments. For a model trained in a few environments (1 or 3), the recurrent cells exhibit clear spatial patterns, while the spatial tuning of units in many (10 and 50) environments becomes less pronounced. With an increasing number of environments, KL divergence Figure 6B and decoding error Figure 6C of the trained model remained at high levels for a longer time period. Even when we trained the models proportionally longer, this effect persisted. Moreover, the 50-environment model showed signs of training convergence, indicating that more training would not considerably improve its training metrics. These findings are consistent with the model not learning to use a general solution. Therefore, the model fails to generalise to an arbitrary number of environments.

We also speculated that finding a general solution might be harder than adapting an existing solution, when presented with unseen environments. To this end, we also trained the model continually in three environments. The results are summarized in Figure S4. Despite a slight increase in convergence after the presentation of each new environment, these results do not provide any evidence supporting reuse. On the contrary, the model catastrophically forgets previously visited environments, which is reflected in the ratemaps becoming flat and noisy (Figure S4D), compared to clear spatial patterns in (Figure 2D).

## Discussion and conclusion

Although a plethora of experimental^2^^,^^4^^,^^5^^,^^6^^,^^7^^,^^8^^,^^37^ and modeling studies^3^^,^^28^^,^^29^^,^^30^^,^^32^^,^^35^^,^^38^ have investigated the contribution of grid cells to path integration, their role remains a subject matter of ongoing debate.^39^ Indeed, we observe that grid cells emerge in the model as a result of learning path integration. This suggests that grid cells are necessary for optimally solving path integration. Similar to Nayebi et al.,^40^ our first surprising result is therefore that grid cells appear relatively unimportant when assessing path integration performance as a function of cell pruning.

Conversely, targeting ensembles of cells that collectively encode a torus has a much higher impact on the model’s path integration performance. Since decoding accuracy rapidly degrades as the toroidal structure breaks, we conclude that the torus manifold is the integral structure that realizes path integration. The cells comprising this structure predominantly share the characteristics of band cells rather than grid cells. Overall, our findings support the converging understanding in neuroscience that ensembles of cells collectively form units of functionality instead of single cells.^11^^,^^41^^,^^42^^,^^43^

Besides toroidal cells, our analysis identified two other clusters of cells that influence the decoding error significantly (clusters with identity 3 and 8 in Figure S1). However, due to their low cell count, we restricted our focus to toroidal cells. Toroidal cells are also more comparable with real grid cells which are found in experiments to form a toroidal structure in their state space.^11^ Interestingly, however, these two clusters contain cells that share the firing characteristics of border-like cells. These border-like cells might serve a range of roles, from facilitating place cell formation as suggested by,^44^ to error correction at boundaries as observed in previous Recurrent Neural Network (RNN) models,^28^ or perhaps even subtler roles such as stabilizing the RNN dynamics in general, or the emergent continuous attractor (torus) specifically. We leave untangling the functional role of these cells to future studies.

Having discussed the functional role of toroidal cells and border-like cells, we are left questioning the role of the remaining cells in the model, including the grid cells. As per the pattern formation theory by Sorscher et al.,^30^ grid cells develop an optimal pattern to produce place patterns, a phenomenon also demonstrated computationally.^31^ This argument might suggest that grid cells primarily facilitate an optimal place-cell decoding operation. Supporting this notion, we see that the decoding error is almost flat in time when pruning grid-like cells (Figure S2) suggesting their role may be less dependent on time. Instead, they may serve a more static role, such as place cell pattern decoding.

Regarding remapping, we expect from experimental evidence that grid cells will remap coherently in both phase and orientation when place cells globally remap.^12^ We assume this also extends to toroidal cells. Figure 4 shows how toroidal cells remap coherently in phase, but retain their orientation between environments. Although unexpected, the zero-remapping in orientation is in accordance with pure phase remapping of grid cells found in response to non-geometric, contextual manipulations of the environment.^45^ Marozzi et al.’s corresponds well with our experimental setup, in which the geometry is kept fixed during remapping. Although less distinct, high grid score cells also show signs of coherent phase shifts and zero-orientation remapping. On the other hand, the low-dimensional projections and persistence diagrams of the high grid score cells show that they do not collectively encode a simple geometry, nor is their collective behaviour coherent across environments.

A mechanistic interpretation of phase-remapping in the CARNN suggests that place cells represent positions in physical space on the torus via the model’s linear encoder layer. Another set of place cells, for example as a result of global remapping, would result in a different set of initial positions on the torus. Because the torus is persistent, those initial positions are on the same torus. Hence, remapping the place cells shifts the spatial pattern of all the cells encoding the torus coherently.

A coherent orientation shift may, on the other hand, be caused by the upstream head direction circuit found in the Subiculum.^46^^,^^47^ In our model, the velocity input models the signals of that circuit. If we assume that the torus in the CARNN encodes a CANN,^3^ the orientation shift amounts to reorienting the basis of the velocity input. Since the head direction cells are anchored to distal cues,^48^ a rotation of the velocity input basis reflects a relative rotation between the internal and physical representation of space. This discrepancy effectively translates into a rotation of the activity pattern of the toroidal cells.

Based on the results of this study we suggest several routes of experimental verification. Experiments could target and prune specific cell types and subsequently test path integration performance, for example using gain control in a virtual maze^5^ or a water maze.^49^ These kinds of pruning experiments (assuming no secondary or off-target effects) could represent a first step toward identifying a causal structure in the mechanism of path integration in the hippocampal trisynaptic circuit. As such, even if grid- or toroidal-cell pruning affected path integration performance through mediation, a deterioration of that performance would still indicate those cell clusters being an essential node in the causal chain for path integration. Homeostatic effects, on the other hand, could lead to a false negative in the sense that path integration was not affected due to redundancy. However, if the MEC homeostatically compensated for eliminating cells in the grid (torus) cluster, compensation might occur on time scales measurable in path integration performance. In this scenario, measuring path integration performance over time might uncover homeostatic effects by showing a surge in path integration error followed by a subsequent decline. If homeostatic effects happened at time scales too fast to measure, they would either be a result of other, identifiable cells within the same cluster, compensating for the loss of cells, or other neuron types in the brain taking over the role of toroidal cells. Another line of experiments would aim at uncovering the contribution of head direction (HD) cells during remapping. The presented results suggest that this cell population directs the rotational shift in grid cells. Inducing a rotation in the HD system could, then, rotate the spatial pattern of grid-like cells.

## Limitations of the study

Prior studies have demonstrated that pattern formation in RNN models trained for path integration can be influenced by factors such as the model type and specific choices of place cell hyperparameters.^40^^,^^50^ In our model, we use the same hyper-parameter settings as Sorscher et al., because we are interested in further studying the grid-like structures that were initially found in their study.^30^ With this, we are fully aware that such structures only exists in a small subset of the hyper-parameter space, as Schaeffer et al. criticized.^50^ However, we do not see this as a limitation, as Sorscher et al. clearly communicate their model assumptions. The toroidal manifold, being a low-dimensional representation of a subset of these patterns will, likewise, be sensitive to these factors. In contrast to that, we do not anticipate the torus to be largely dependent on the remapping mechanism in light of its structural persistence despite our substantial remapping method. Potential future research could investigate the robustness of these low-dimensional toroidal representations, exploring the impact of variations in both the model architecture and the hyperparameters of the place cell basis.

In the MEC, grid cells are organized in modules according to their spacings along the dorsoventral axis. However, our analysis of the spacing of cell patterns revealed that their distribution is unimodal. Consequently, we could not detect any partitioning into multiple modules. This finding is in accordance with ref.^50^ but contradicts refs.^29^^,^^51^ Schaeffer et al.^50^ argue that the scale of the grid cells follows the scale of the place cells. Thus, implementing a spatial organization of place cells with varying scales, similar to that in the hippocampus,^52^^,^^53^ offers a promising approach to introduce multiple modules. The presence of multiple modules is desirable because it enables the study of how different modules remap relative to each other. The purpose of multiple modules is thought to improve spatial encoding precision and to expand the area of unique spatial encoding, especially when considering modules with distinct orientations (see refs.^54^^,^^55^). The concept of RNNs operating at varying scales is complex and may be achieved with different methods. For example, different spatial scales may be indirectly achieved by using a network with several distinct temporal scales (akin to a clockwork RNN as suggested by ref.^56^), alterations in the speed of the velocity input (an idea considered in ref.^35^), or even variations in the scales of place cell tuning curves (as described in ref.^50^ and used in ref.^57^).

One potential limitation of the presented model is the global place cell remapping in the form of complete random resampling of place cell centers for different environments. While this aligns with experimental findings of global remapping in place cells being completely uncorrelated in different environments,^13^^,^^58^ this method represents a rather strong transformation for multiple reasons. For one, only one-third of randomly selected place cells tend to be active in a particular environment.^13^^,^^59^ Second, from a normative perspective, it seems unlikely that the brain implements place cell remapping with no underlying structure, contrary to our random resampling assumption. The latter point is supported by findings in ref.^38^, where place cell firing field locations were found to be correlated with grid cell lattice points. However, our remapping analyses aim to capture the phase and orientation remapping phenomena that are observed in grid cells. As a consequence, they are independent of the specific place cell remapping formulation we choose. From experimental results, we expect the largest effect in grid cell remapping when place cells are globally remapped, which is why we chose this form of remapping. It is, however, worth noting that the symmetry assumptions (hexagonal unit cells in Figure 4, −15 to 15° orientation shifts) are related to ideal grid patterns, and hence may be less informative for non-symmetric patterns. Regardless, we consider this relevant information, as it highlights (dis)similarities between the empirical pattern and an ideal grid pattern.

Another possible indication that the current remapping formulation is too strong, or the model too simple, is that the model is not able to generalize to many environments. This limitation is evident in Figure S6 (extending Figure 6 with more ratemaps, clustering and low-dimensional representations), where we clustered cells from the 10ME network, revealing only faint spatial patterns. Moreover, low-dimensional projections failed to discern any straightforward geometric connections. However, we also realize that this discrepancy is probably in part due to constraints we impose by the choice of model architecture. In particular, a linear map from the place cell basis into the recurrent layer may not be sufficient to translate a random sampling of place cell locations into a shift in phase and orientation on a continuous attractor manifold. Therefore, potential future approaches include (1) considering more coherent remapping schemes and (2) extending the model to account for the extent of the current remapping mechanism. Particularly, accounting for the lateral entorhinal cortex (LEC) in the encoder and decoder structure could incorporate explicit context signals regarding environment identity that the model might benefit from, similar to the Tolman-Eichenbaum machine (TEM).^38^

When training the model continually, catastrophic forgetting is unlikely to be avoided without additional weight-preserving regularization strategies. On the other hand, if a set of tasks contains a sufficiently similar solution, and the model is primed for learning such a solution, continual learning might actually be solved because of how well-posed the task and the model are. Navigation in multiple environments may be such a task because the properties of space and path integration are preserved across environments, which motivated us to produce the results in Figure S4. Learning continually, then, should ideally lead to slow learning for the first environment, because path integration must be learned from scratch, while new environments can be learned quickly because the model can rely on the existing prior solution, and only have to learn the new environment signatures. While such a sequential learning mechanism may be well suited for a continual learning experiment, our findings show that the model catastrophically forgets. Finding methods that enable the reuse of a path-integrating structure could be a fruitful direction for future studies.

Reciprocal connections between the hippocampus and the MEC suggest a bidirectional relationship.^60^ This complex interaction might be essential for place cells to inform grid patterns and remapping.^20^^,^^26^ In our model, place cells and remapping are fixed to a specific functional form. During inference, this setup mimics the connectivity of the trisynaptic loop, where place cells from the hippocampus are influencing (initialising) the upstream MEC cells, which again feed onto the hippocampus, not dissimilar from the Hippocampal drive in many of the CANN models; see ref. ^61^ However, during learning, the analogy breaks down due to the fixed place cell maps. Therefore, a possible extension of the current model could be to relax the strict functional form of the place cells and the remapping, allowing not only the place cells to influence the pattern formation of the recurrent cells but the recurrent cells to also influence the pattern formation and remapping of the place cells. Such a modelling approach could be a promising direction for future research endeavours. It i, however, beyond the scope of this study as it requires a complete reformulation of the labels and training objective.

## STAR★Methods

### Key resources table

**Table:** 

REAGENT or RESOURCE	SOURCE	IDENTIFIER
**Software and algorithms**		

UMAP v0.5.2	L. McInnes et al.^36^	https://github.com/lmcinnes/umap
Ripser 0.6.1	N. Saul and C. Traile.^62^	https://ripser.scikit-tda.org/en/latest/index.html
PyTorch v1.13.0+cu117	A. Paszke et al.^63^	https://pytorch.org
DBSCAN (from scikit-learn)	M. Ester et al.^64^	https://scikit-learn.org/stable/modules/generated/sklearn.cluster.DBSCAN.html
Scikit-learn v1.0.2	F. Pedregosa et al.^65^	https://scikit-learn.org/stable/index.html
Matplotlib v3.7.1	J. D. Hunter^66^	https://matplotlib.org
Scipy v1.9.0	P. Virtanen et al.^67^	https://scipy.org
Spatial-maps	Lepperød et al.,^68^ Christensen et al.^69^	https://github.com/CINPLA/spatial-maps
Grid score algorithm	Banino et al.^29^	https://github.com/deepmind/grid-cells/blob/master/scores.py
Original code: Trajectory generation	https://doi.org/10.5281/zenodo.8318064	https://github.com/bioAI-Oslo/rat-simulator
Original code: RNN model and analysis	https://doi.org/10.5281/zenodo.8318009	https://github.com/bioAI-Oslo/emergent-grid-cells

### Resource availability

#### Lead contact

Further information and requests for resources should be directed to and will be fulfilled by the lead contact, Mikkel Elle Lepperød (mikkel@simula.no).

#### Materials availability

This study did not generate new unique reagents.

### Method details

#### Generating dataset trajectories

The dataset used to train and test the model consisted of simulated place cell activity along 2D random walks in a square enclosure. To simulate trajectories, we created a simple framework for generating random walks in 2D geometries. The toolkit is openly available from https://github.com/bioAI-Oslo/rat-simulator. In this work, the statistics of the random walker follow that of the random walker used by ref.^30^

There are two objects interacting in the random walk: an agent that performs the walk, and an environment that constrains the walker’s movement inside some geometry. In this work, we considered an environment consisting of a square box of size 2.2×2.2. The walls of the environment featured soft boundaries with margins of 0.03 to the chosen box size.

Both the initial head direction and position were sampled from uniform distributions in the square environment, in any direction. Consecutive positions in the walk were computed by integrating the initial position with generated Cartesian velocities. The integration time constant was set to τ=0.02. Velocities were drawn by sampling a speed and head direction at every simulation step. Speed values were drawn from a Rayleigh distribution with a scale parameter set to b=2⋅0.13π. Head directions were sampled from a normal distribution with mean at the previous head direction and standard deviation σ=2⋅5.76. In addition to the initial location, all trajectories featured 20 timesteps.

#### Positions in place cell basis

The initial position input to the RNN and the training targets were encoded in place cell coordinates, following the approach in ref.^30^ Each place cell was modeled as a difference of two softmax functions, according to

(Equation 1) $$p_{i}\left(\underline{x}\right)=\sigma_{i}\left({\underline{z}}_{1}\right)-\sigma_{i}\left({\underline{z}}_{2}\right)$$

where

$$z_{j1}=-\frac{∥\underline{x}-{\underline{\mu }}_{j}{∥}_{2}^{2}}{2s_{1}^{2}}\;\text{and}\;z_{j2}=-\frac{∥\underline{x}-{\underline{\mu }}_{j}{∥}_{2}^{2}}{2s_{2}^{2}}$$

with $i,j=1,2,\ldots ,N_{p}$. Here, $s_{1}=0.12$ and $s_{2}=2s_{1}$ are parameters shared across all cells, setting the tuning width of each place cell. ${\underline{\mu }}_{j}\in R^{2}$ is the center coordinate of place cell *j*, $\underline{x}\in R^{2}$ is the current Cartesian position of the agent, and $\sigma_{i}$ denotes the *i*th component of the Softmax function over all $N_{p}$ place cells. Center locations were sampled randomly within the square environment, according to a uniform distribution. Finally, the place cell ensemble activity was shifted and normalised to be positive everywhere and sum to one at every location, enabling training using cross-entropy loss.

Decoding from place cell activity to Cartesian coordinates was done using a center of mass approach. First, the three most active cells at a given location were selected. Then, the decoded position was taken to be the spatial average of the center locations of these three units.

#### Simulating place cell global remapping

Different environments were modeled in terms of the activity of an ensemble of simulated place cells. In each environment, the center locations of all $N_{p}$ place cells were randomly permuted, so that ensemble activities were independent across environments. Concretely, the center location of each place cell was sampled from a uniform distribution in a given environment, while the functional form of the unit was maintained, and given by Equation 1. See Figure 1A for an example.

#### The model

The neural network architecture was identical to the architecture used by Sorscher et al. which consisted of a simple RNN layer, and two dense layers. The first dense layer featured $N_{g}=4096$ units, encoding the initial position of a trajectory into an initial RNN state. The initial position was provided as a vector of all $N_{p}=512$ place cell activities, according to (Equation 1). In other words,

(Equation 2) $${\underline{g}}_{0}=W_{e}{\underline{p}}_{0}$$

where ${\underline{g}}_{0}$ is the initial state of the RNN, $W_{e}\in R^{N_{g}\times N_{p}}$ is a matrix of encoder weights, and ${\underline{p}}_{0}\in R^{N_{p}}$ the initial place cell ensemble activity. Consequently, the RNN featured $N_{g}=4096$ recurrent units, each equipped with a ReLU activation function with no added bias. At each time step, the RNN received a two-dimensional Cartesian velocity input, as described in generating dataset trajectories. The state of the RNN at a given time *t* may therefore be written as

(Equation 3) $${\underline{g}}_{t}=\text{ReLU}\left(W_{g}{\underline{g}}_{t-1}+W_{v}{\underline{v}}_{t}\right)$$

where $W_{g}\in R^{N_{g}\times N_{g}}$ is the recurrent weight matrix, while $W_{v}\in R^{N_{g}\times 2}$ is a matrix of input weights. The final dense layer consisted of $N_{p}=512$ output units, followed by a Softmax function, which decoded RNN states to place cell ensemble activity at each time point. Thus, the predicted place cell activity may be written as

(Equation 4) $${\hat{\underline{p}}}_{t}=\underline{\sigma }\left(W_{d}{\underline{g}}_{t}\right)$$

where $W_{d}\in R^{N_{p}\times N_{g}}$ are the decoder weights, and $\underline{\sigma }$ the vector-valued Softmax function. All weight matrices were initialised using Xavier uniform initialisation^70^ with unity gain.

#### Loss function and training details

As in Sorscher et al.,^30^ the loss function features two terms: a cross-entropy term between the predicted and true place cell activity, and an L2-regularisation. For a single trajectory of length *T*, the loss may be written

(Equation 5) $$L=-\frac{1}{T}\sum_{t=1}^{T}\sum_{i=1}^{N_{p}}p_{i}\left({\underline{x}}_{t}\right)\;\ln\;{\hat{p}}_{it}-\lambda ∥W_{g}{∥}_{F}^{2}$$

where $p_{i}\left({\underline{x}}_{i}\right)$ is the activity of the *i*th label place cell, at time *t*, while ${\hat{p}}_{it}$ is the corresponding model prediction given by Equation 4. $∥\cdot {∥}_{F}$ denotes the Frobenius norm, while λ is a hyperparameter penalising the magnitude of the recurrent weights, which was set to $\lambda =10^{-4}$.

We use the KL divergence for visualising training convergence rather than the cross entropy. This is because the optimum for the KL divergence is at zero, whereas the cross entropy optimum is at the entropy of the label distribution.

We trained models in both multi-environment and continual learning settings. The network was trained to minimise (Equation 5) using batched gradient descent. For multi-environment training, each mini-batch contained an equal amount of training data from each environment. During continual learning, all the data and labels from one environment were exhausted before presenting data and labels from a new environment. We trained the multi-environment model in 3, 10 and 50 environments. For the continual learning task, the network was trained in three environments.

A given model was trained for a total of $10^{5}$ mini-batches multiplied by the number of unique environments visited in the training data, using the PyTorch^63^ Python library. All models were trained using the Adam optimiser^71^ with default parameters, except for the learning rate which was set to $10^{-4}$. The model was trained on mini-batches of size 200, and the trajectory length was set to T=20.

#### Pruning

To understand the contribution of individual cell types to path integration performance, we adopted pruning strategies. This approach involved selective elimination of weights in the model trained across three distinct environments. We restricted pruning to the recurrent layer of the network. Concretely, we inactivated a unit by selecting all its incoming weights and setting them to zero. This encompasses both recurrent incoming connections and velocity input weights for a recurrent unit. By pruning all incoming weights to a cell, it becomes effectively inactive, considering there is no network noise.

We carried out three distinct pruning experiments (Figure S1 and S2 which are related to Figure 3). In all scenarios, the decoding error is calculated as outlined in positions in place cell basis, and averaged over mini-batches of 200 navigation examples drawn from the three learned environments. To enhance the robustness of our findings, each experiment was performed 30 times, producing median curves and median absolute deviation (MAD) error shading. The graph depicting pruning error as a function of the number of pruned units (Figure 3) presents five unique models. For every model, the decoding error is calculated for the 20th timestep.

We selected this particular timestep because, as time progresses, path integration errors become more substantial due to their cumulative nature, and the model is trained to minimise errors up to the 20th timestep. The ’Full model’ and ’Full Untrained model’ in Figure 3 serve as reference points, providing upper and lower limits on decoding error, respectively, and are unaffected by the pruning. In the ’Random Pruning’ model, cell indices are incrementally chosen at random, without replacement, from the 4096 available recurrent cells.

To prune grid cells, we rank the recurrent cells based on their highest cumulative grid score across all three environments. We included the top 604 grid cells, bringing the ensemble size in line with that of the torus ensemble. The pruning of grid and torus cells is carried out in increments of 10 cells, randomly selected from the set of 604 indices without replacement.

The experiment involving the pruning of all clusters in relation to the number of cells (see Figure S1) followed a similar process to the pruning of grid and torus cells. However, the variable number of cells across different clusters means that not all curves extend to the full 360-unit pruning interval.

To study the time dependence of the decoding error resulting from pruning (Figure S2), pruning was performed in a similar manner as before. However, for these trials, we fixed the number of pruned cells to 100 and evaluated the model’s decoding error at every timestep instead.

#### Synthetic grid cells

We model synthetic grid cells to compare learned remapping with different idealised remapping scenarios. We follow Solstad et al.^17^ and generate grid cells through constructive and destructive interference of three plane waves with unit wave vectors ${\underline{k}}_{j},j=1,2,3$ oriented at 60° to one another. This can be written as

(Equation 6) $$g_{i}\left(\underline{x}\right)=\text{ReLU}\left(\sum_{j=1}^{3}\cos\left(2\pi fR{\underline{k}}_{j}\cdot \left(\underline{x}-{\underline{x}}_{i}\right)\right)\right)$$

where the subscript *i* indexes a particular grid cell. R∈SO(2) is a rotation matrix, that sets the orientation θ of the pattern, while *f* is its spatial frequency. Finally, $\underline{x}\in R^{2}$ is a spatial coordinate and ${\underline{x}}_{i}\in R^{2}$ is the pattern phase.

To simulate grid modules, grid cells within a common module were set to have the same spacing and orientation, but phases were sampled uniformly within the unit cell of the pattern. Remapping between environments within a module was by coherently shifting phases and orientations. For all modules, the grid frequency was fixed to f=1/0.838, which was the learned frequency of the high grid score cells in the CARNN model, found by the method outlined in grid spacing.

We simulated five different grid cell modules to show four different remapping scenarios: (in)coherent phase and (in)coherent orientation remapping. The first module served as a default model to measure remapping against. The four other modules were identical to the default model apart from one of the following: All cells were (i) coherently phase-shifted by (−0.2,−0.2), (ii) coherently reoriented by −5 degrees, (iii) (incoherent) random uniformly resampled in the unit cell (iv) (incoherent) randomly and uniformly rotated.

#### Clustering and grid module identification

To investigate whether the units in the recurrent layer are self-organised into clusters resembling grid cell modules, we employed a clustering procedure similar to the one proposed by Gardner et al. However, rather than perform clustering based on standard spatial autocorrelations, we instead consider rotational autocorrelations.

First, activities were generated by running the network on a set of 10000, 20-timestep trajectories. Using the generated trajectories, 32×32 ratemaps were formed. For these ratemaps, only the final 10 trajectory timesteps were included to avoid spurious initial network states. In the subsequent analysis, we also excluded units with zero activity all over space (n=954 units). In addition, we found that a small number of units had substantially greater mean spatial firing rates, and exhibited weak spatial tuning. We subsequently removed these bias-type units before clustering, by excluding units with a mean spatial firing rate above the 99.75-percentile (n=11 units). After exclusion, we were left with 3131 units, which were subjected to clustering.

To cluster units, we first computed rotational autocorrelograms. This was done by first slicing out the outermost pixels of a given ratemap, forming an approximate disk of radius 16 pixels. Each disk was subsequently normalised to have zero mean and unit variance. Then, the Pearson correlation coefficient between a rotated disk and the unrotated version was computed for $N_{\theta }=360$ incremental rotation values ranging from −π to π. Thus, for each 32×32 bin ratemap, a vector of $N_{\theta }$ rotational correlations was formed.

To cluster cell types based on their rotational symmetry, we stacked each rotational autocorrelogram into an $N_{g}\times N_{\theta }$ matrix. We then applied UMAP^36^ to this matrix, projecting a given unit’s autocorrelogram into two dimensions. For dimensionality reduction, UMAP hyperparameters were *metric* = ’*Euclidean*’, *n_neighbours* = 20, *min_dist* = 0.05, and *init* = ’*spectral*’ with other parameters set to their default value. Finally, the resulting 2D pointcloud was subjected to clustering using DBSCAN.^64^ For DBSCAN, *min*_*samples* = 30 was used, while other parameters followed the scikit-learn defaults.^65^ The resulting clusters (Figure S1) contained units that had similar autocorrelograms and, thus, similar rotational symmetry. We find that this procedure produced clusters containing units with distinct spatial responses, that were also aligned with biological cell types. For example, certain clusters only contained band-like units, while others only displayed grid-like spatial representations.

#### Cluster refinement

The clusters identified by the automated procedure detailed in clustering and grid module identification can be further classified qualitatively. In particular, we found that three clusters (2, 12 and 15, in Figure S1) contained units with representations similar to band cells. Notably, the directions of these bands appeared to be oriented at approximately 60-degree angles relative to each other. We therefore manually grouped these clusters into a collective group, which we termed the ’torus ensemble’.

To validate this observation, we further separated torus units into three predetermined clusters using K-means clustering. This unsupervised learning method was applied to Pearson correlation coefficients computed between pairs of cell autocorrelograms. To visualise this clustering, we provide examples of randomly selected ratemaps from each cluster in Figure S5. These examples highlight the distinctive characteristics of each subgroup within the torus ensemble. Note that all visualizations, for all analyses were performed using the Matplotlib Python library.^66^

#### Low dimensional projection of cell clusters

To visualise low-dimensional structures in the learned representations of the network, we applied the same two-stage dimensionality reduction procedure used in ref.^11^ First, ratemaps of recurrent unit activity were flattened and stacked into a matrix, so that each column contained the spatial responses of a single unit, while a row represented the ensemble activity at a particular spatial bin. PCA was then performed on this matrix, projecting the $N_{g}$-dimensional network activity down to six principal components. Following PCA, an additional UMAP dimensionality reduction was performed, reducing the dimensionality to three. A point in the resulting three-dimensional point cloud thus represents the ensemble activity of the included network units at a particular spatial bin. For this visualisation procedure, non-default UMAP hyperparameters were *n*_*components* = 3, *min*_*dist* = 0.8, *n*_*neighbours* = 4000, *metric* = ’*Euclidean*’, *init* = ’*spectral*’.

### Quantification and statistical analysis

To extract autocorrelogram peaks for determining grid score, orientation, spacing and phase shift, we used utilities from the Spatial Maps (Available from https://github.com/CINPLA/spatial-maps) toolkit.^68^^,^^69^

#### Ratemaps

Ratemaps were created by running a network on a collection of simulated trajectories (random walk) and aggregating unit activities from the model during inference on those trajectories. Then, the ratemap value at a particular spatial bin was computed as the average unit activity over all visits to that location. Unless otherwise specified, all ratemaps had a spatial resolution of $N_{s}\times N_{s}=64\times 64$ and were made using samples aggregated over all 20 timesteps from 5000 simulated trajectories.

#### Grid score

The grid score is computed following Banino et al.^29^ This process can be summarised in three main steps: (i) A 2-dimensional spatial autocorrelation is performed on a given ratemap. (ii) An annulus mask is applied to eliminate the central peak and the peripheral regions of the autocorrelogram. (iii) The resulting data are then subjected to autocorrelation at 60 and 90-degree rotations. The grid score is the difference in correlations at 60° and 90°. Therefore, high correlations at 60° and low correlations at 90° result in high grid scores.

#### Grid phase shift

To calculate the phase shift between two grid cell patterns, we first computed the two-dimensional correlation between their ratemaps. Subsequently, we determined all local peaks in the cross-correlogram and selected the peak closest to the center. The coordinate of this peak relative to the center of the cross-correlogram determined the phase-shift vector. For an ideal grid pattern (see synthetic grid cells), phases are unique up to the unit cell defined by the pattern, which is a hexagon with a radius of two-thirds of the pattern frequency *f*, defined in Equation 6.

When plotting phase shifts, we include a kernel density estimate using a Gaussian kernel. All kernel density estimates were calculated using the *Gaussian_kde* method from SciPy^67^ with default (Scott’s Rule) kernel bandwidth and n=604 and d=2.

#### Grid orientation shift

Calculating the grid orientation shift between two ratemaps involves several steps. Initially, we computed the autocorrelogram for both ratemaps and normalised the sum of each to one. Subsequently, we rotated one autocorrelogram with respect to the other, in one-degree increments. We then selected the largest overlapping disk between the square autocorrelograms. Finally, we computed the Pearson correlation between the original and rotated autocorrelogram, for each rotation. The grid orientation shift was then selected as the rotation that yielded the highest correlation. For 30-degree wrapped orientation shifts, we first shift orientations by 15° before computing the 30-degree modulo, followed by recentring.

#### Grid spacing

To estimate the grid spacing from a grid cell ratemap, we start by identifying peaks in the ratemap autocorrelogram. We then identified the isodistant points closest to the origin of the autocorrelogram. Isodistant here refers to peaks that are distributed equidistantly from the origin. Concretely, we detect these points by first computing the difference in centre-distance between neighboring peaks, sorted by their distance to the origin. Isodistant points will exhibit small differences, while non-isodistant peaks will show large (outlier) differences in centre-distance. We find these jumps as two standard deviations from the mean distance difference. The points prior to the first jump determine the isodistant points closest to the origin. We then calculated the median distance of these subselected peaks. Together, this forms a more robust method for estimating the grid spacing than one obtained using, e.g., a single grid vertex. The grid spacing is inversely related to the pattern frequency *f*, as in Equation 6, thus providing a measure of the frequency of repeating elements in the pattern, denoted as 1/f.

#### Topological data analysis

To verify that the network activity resided on a low-dimensional manifold, we investigated the persistence of topological features in the activity of the network by persistent homology. Concretely, we computed persistence diagrams for dimensionality-reduced network activity, using the Ripser and Persim Python libraries.^62^ Analyses were performed for units that were classified as being band-like, and grid-like (see clustering and grid module identification for details).

Low dimensional network activity was computed by applying PCA to ratemaps of network activity, reducing the dimensionality down to six principal components. Concretely, $N_{s}\times N_{s}$ ratemaps for all units were stacked into a matrix with $N_{g}$ columns, and PCA was applied to the columns of this matrix. Thus, the resulting representation consisted of a pointcloud of $N_{s}^{2}$ points in a six-dimensional space, representing a low-dimensional representation of the full network activity at a particular spatial bin. For all topological analyses, the six-dimensional pointcloud was further downsampled using the procedure proposed by Gardner et al. This was done to reduce the impact of spurious activity, as persistent homology can be sensitive to outliers.^11^

Briefly, the approach involves assigning each sample a membership strength in its local neighborhood, corresponding to the first step of UMAP.^36^ Then, downsampling is performed so that the resulting pointcloud consists of the $N\leq N_{s}^{2}$ points with the strongest average neighborhood membership. The membership strength was computed as

$$m_{i,j}=a_{i,j}+a_{j,i}-a_{i,j}a_{j,i}$$

where

$$a_{i,j}=e^{-\frac{d_{i,j}}{\sigma_{i}}}$$

is the similarity between sample *i* and its nearest neighbor number *j*, while $d_{i,j}$ is the cosine distance between said points. Note that $\sigma_{i}$ was chosen so that $\sum_{j=1}^{k}a_{i,j}=\log_{2}\;k$ approximately, with *k* being the number of nearest neighbors.

For downsampling, the number of nearest neighbors was k=1000. Finally, persistence diagrams were computed using the low-dimensional, downsampled point clouds. For all persistence analyses, the final point cloud contained N=3000 points, the metric was taken to be Euclidean, while the coefficient field was p=47 and the number of greedy permutations $n_{perm}$ was 500.

## Data Availability

• Data was simulated using a custom trajectory generator: https://github.com/bioAI-Oslo/rat-simulator • All original code has been deposited at https://github.com/bioAI-Oslo/emergent-grid-cells and is publicly available as of the date of publication. DOIs are listed in the key resources table. • Any additional information required to reanalyze the data reported in this paper is available from the lead contact upon request.

## Supplemental information

Document S1. Figures S1‒S9
