# Supplementary material for: Coherently remapping toroidal cells but not Grid cells are responsible for path integration in virtual agents
Source: iScience. 2023 Sep 30;26(11):108102. doi: 10.1016/j.isci.2023.108102 (PMC10589895; doi:10.1016/j.isci.2023.108102)
Supplement: Document S1. Figures S1‒S9 [file mmc1.pdf]

iScience, Volume *26*

Supplemental information

Coherently remapping toroidal cells but not Grid cells are responsible for path integration in virtual agents

Vemund Schøyen, Markus Borud Pettersen, Konstantin Holzhausen, Marianne Fyhn, Anders Malthe-Sørenssen, and Mikkel Elle Lepperød

Supplementary information

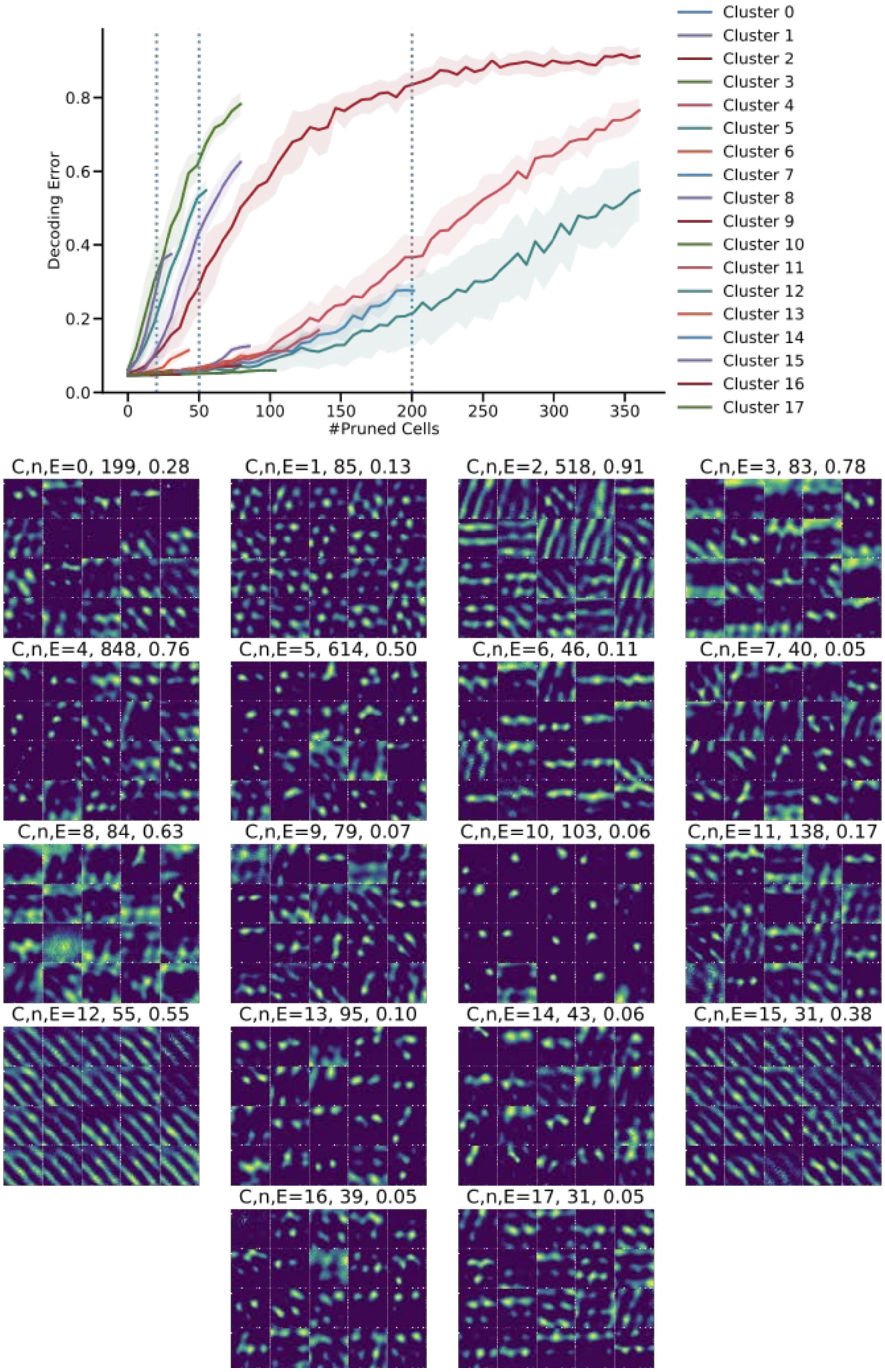

Figure S1: **Cell clusters and their impact on path integration, related to Fig. 3**. The graph in the top row shows the pruning error as a function of the number of cells pruned. Curves and error shadings are given as median ± median absolute deviation. The following rows display a random selection of 20 ratemaps from each cluster. The titles indicate the cluster identity "C", the number of cells "n" in the cluster, and the maximum median error "E" from pruning the cluster.

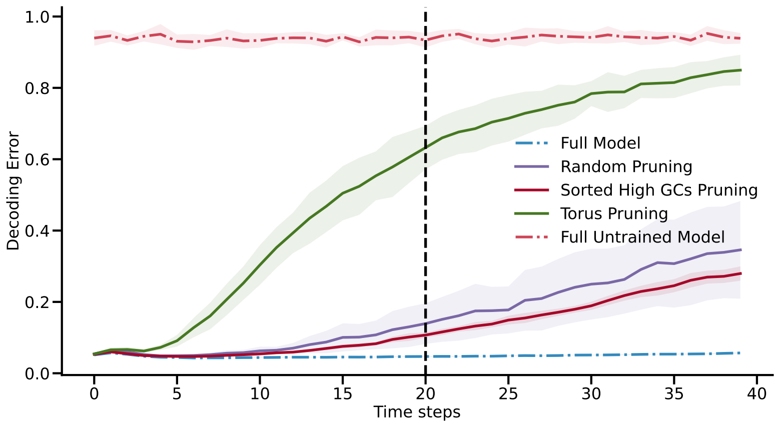

Figure S2: **Time pruning, related to Fig. 3**. Decoding error when pruning 100 cells from different categories as a function of time steps. Curves and error shadings are given as median ± median absolute deviation.

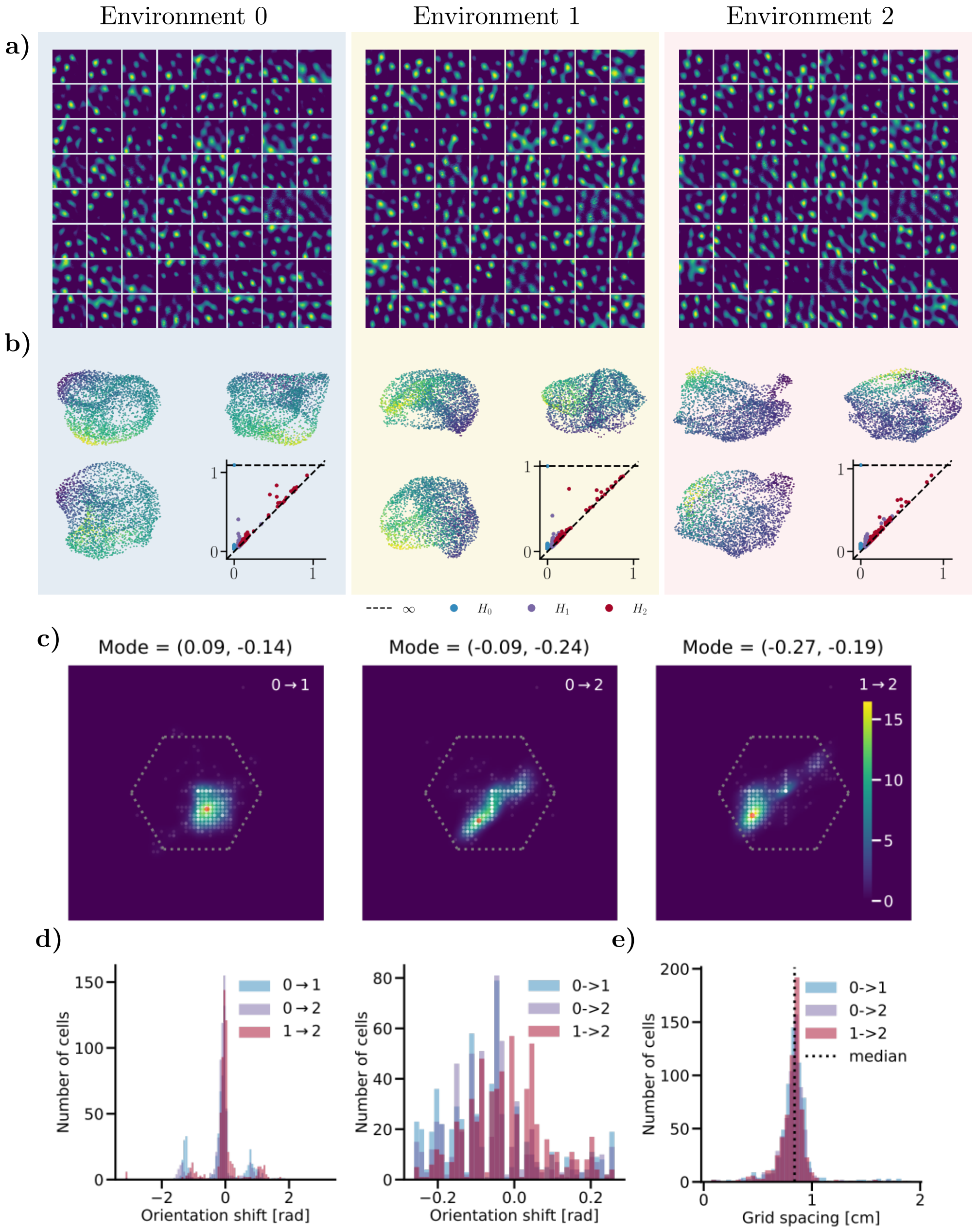

Figure S3: **High grid scoring cells in multiple environments, related to Fig. 4. a)** ratemap examples in the three environments **b)** the low-dimensional projection and persistence diagrams. **c)** phase shift between environments. **d)** orientation shift between environments. **e)** estimated grid spacing.

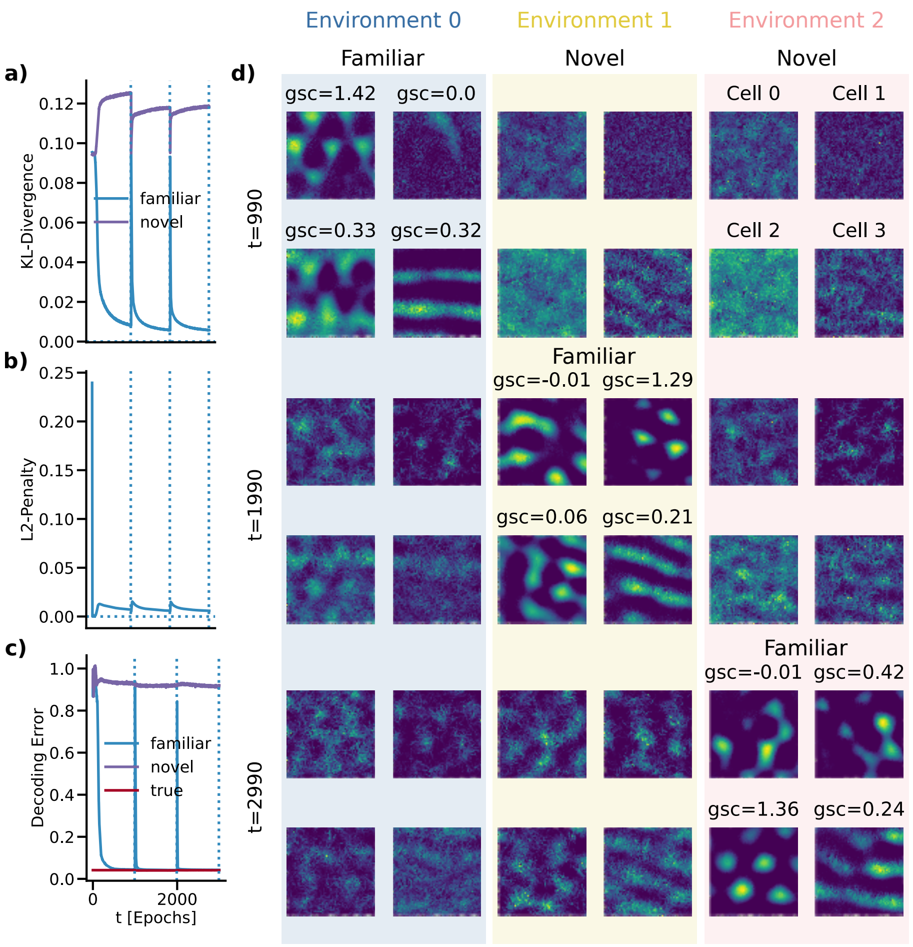

Figure S4: **Sequentially training the model in multiple environments allows the model to adapt to new environments, related to Fig. 6. a)**: KL divergence versus training time t (in epochs) measuring the discrepancy between the predicted and label place cell population activity. **b)**: L^2 weight penalty during training. **c)**: Average decoding error, i.e. the Euclidean distance between predicted and estimated true position during training. **d)**: Firing fields of 4 selected neurons in the recurrent layer of the model. Firing fields are shown for three distinct time points, corresponding to training saturation in different environments (i) $t = 990$, environment 0 (ii) $t = 1990$, environment 1 (iii) $t = 2990$, environment 2. At each time point, the firing fields of the same units evaluated in the other environments is also inset.

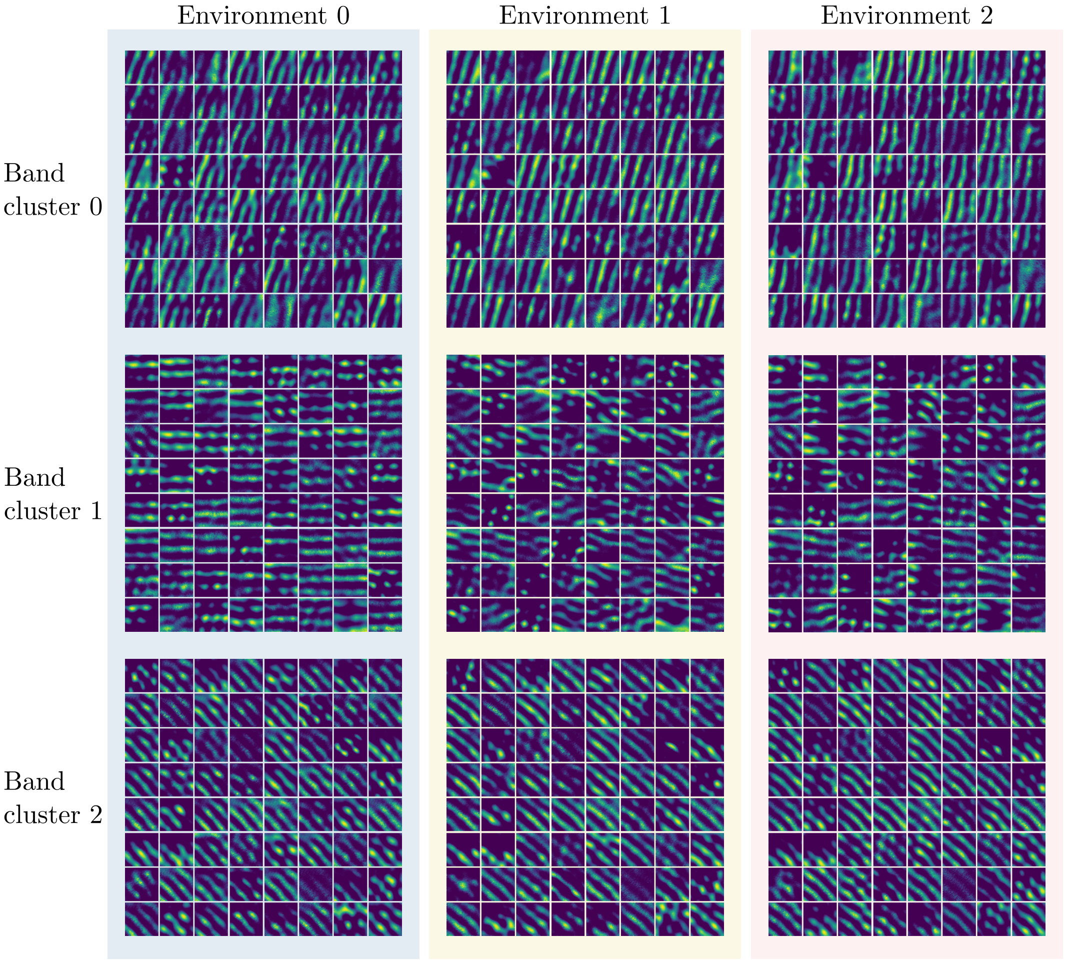

Figure S5: **Band-cells form three clusters at 60 degree offset, related to Fig. 5.** Example ratemaps from the torus cluster after KMeans clustering ($K = 3$) show a clear separation of band-like cells with 60-degree relative orientations in the three environments.

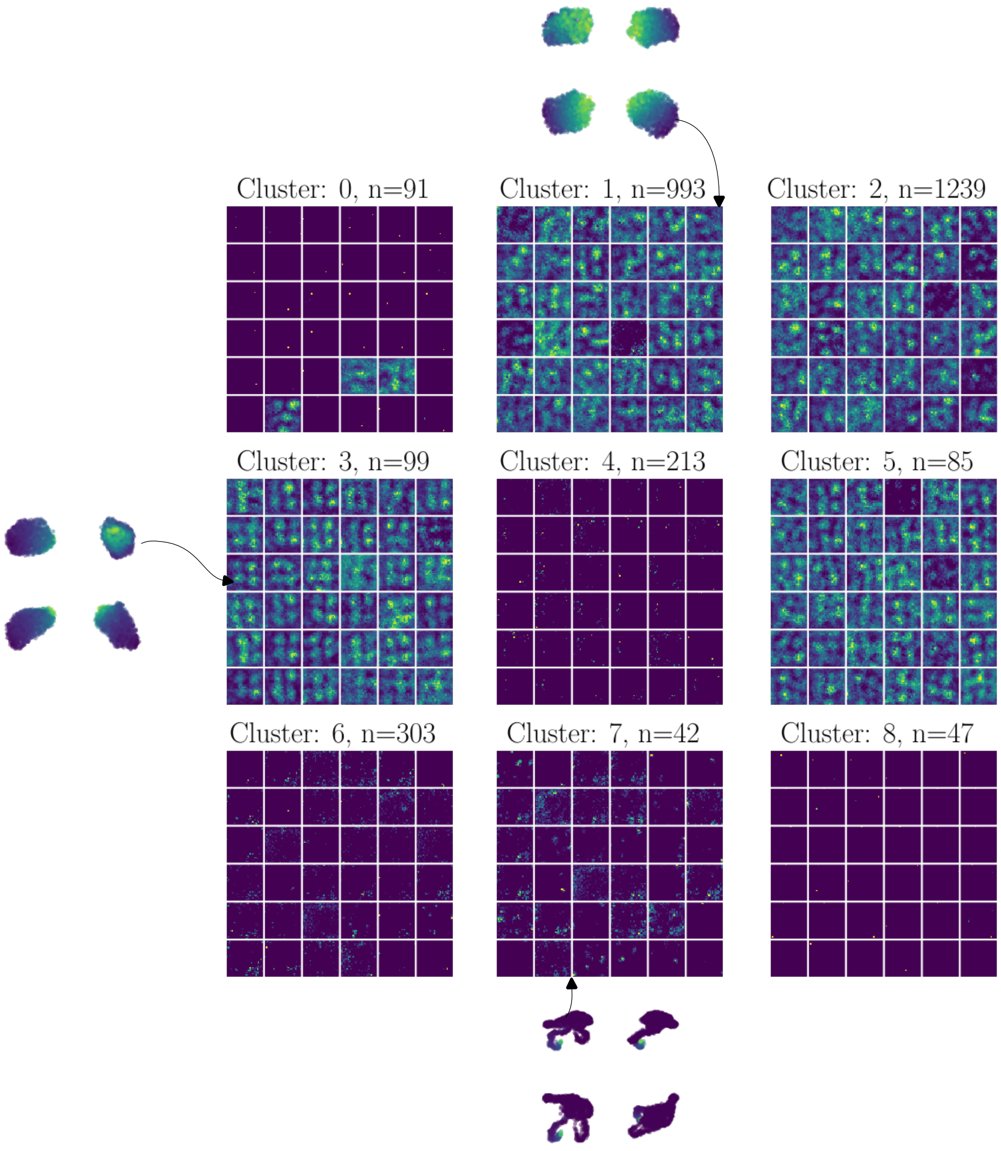

Figure S6: **Clusters and low-dimensional representations of cells from the 10ME model, related to Fig. 6**

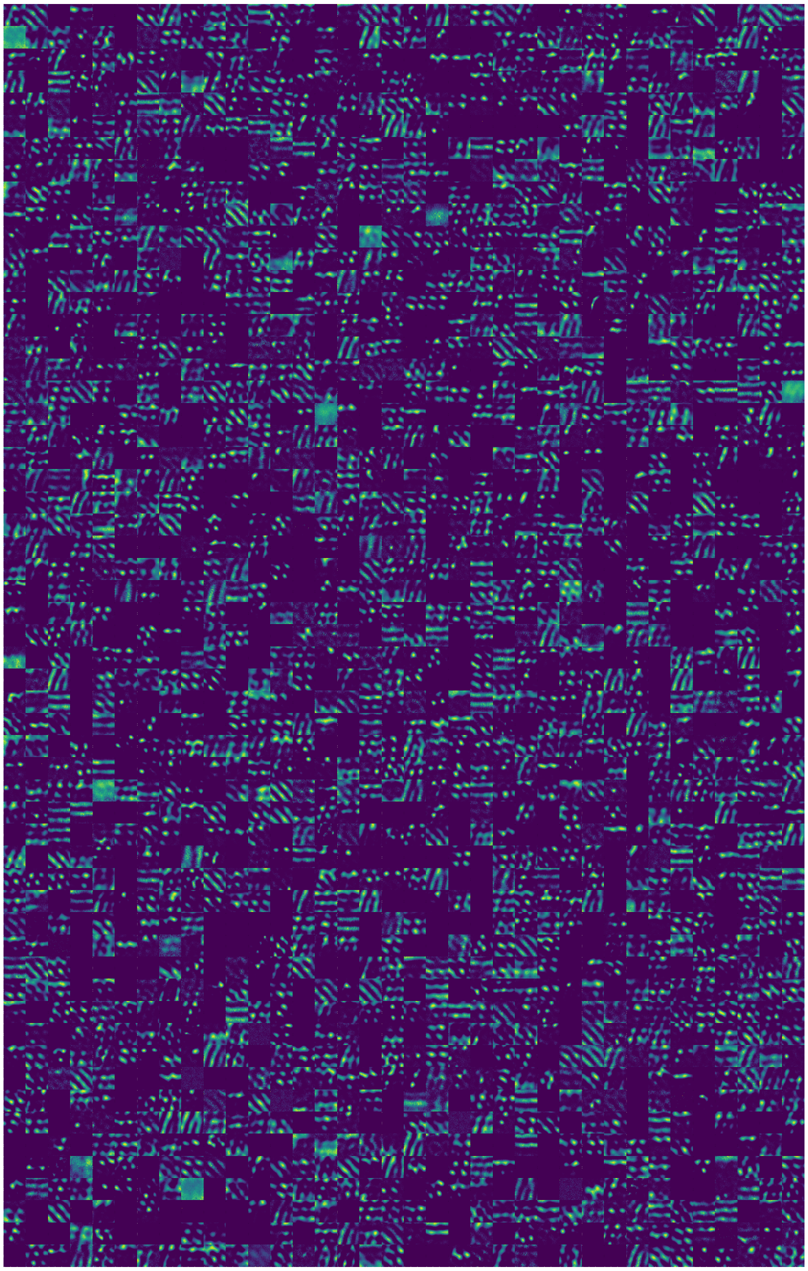

Figure S7: **2052 ratemap examples from familiar environment Environment 0, related to Fig. 2**. Each ratemap in a given position is comparable to the ratemaps from familiar environment Environment 1 (fig. S8) and Environment 2 (fig. S9) (identical cells, different environment).

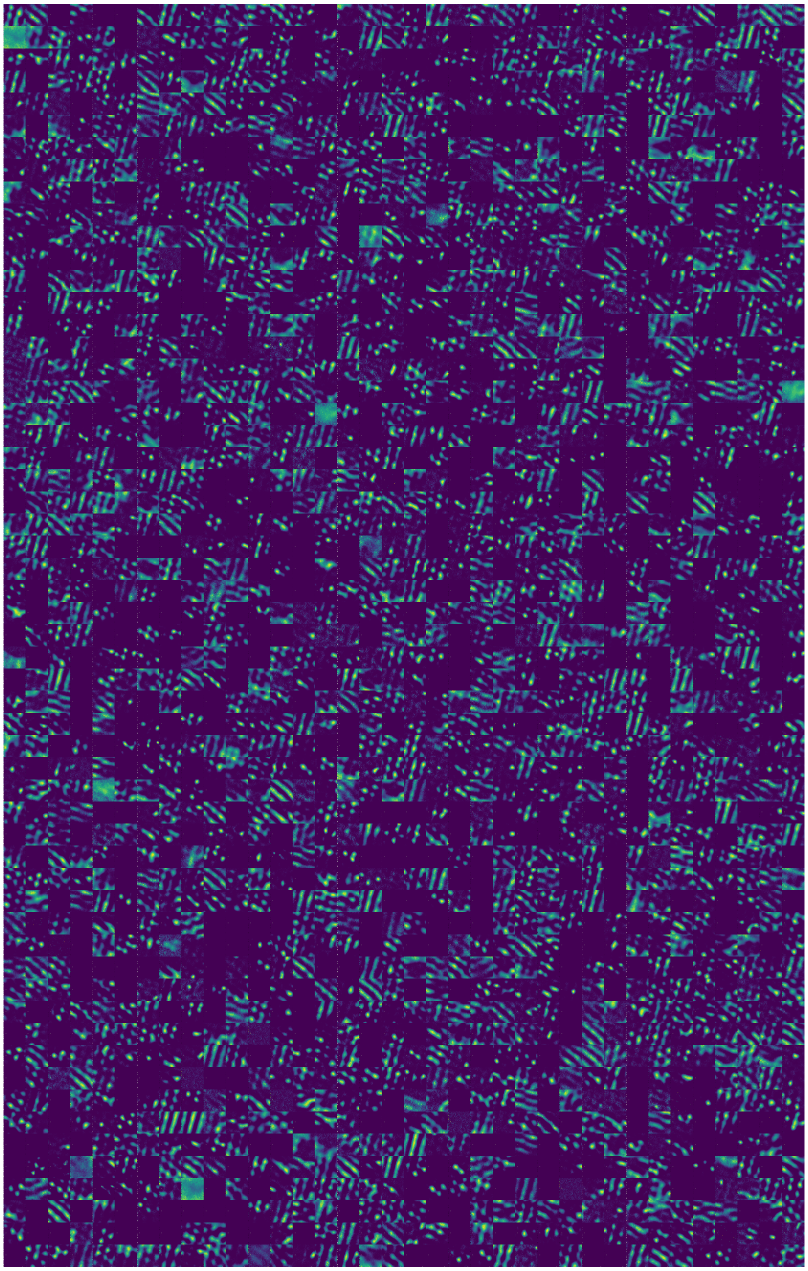

Figure S8: **2052 ratemap examples from familiar environment Environment 1, related to Fig. 2**. Each ratemap in a given position is comparable to the ratemaps from familiar environment Environment 0 (fig. S7) and Environment 1 (fig. S9) (identical cells, different environment).

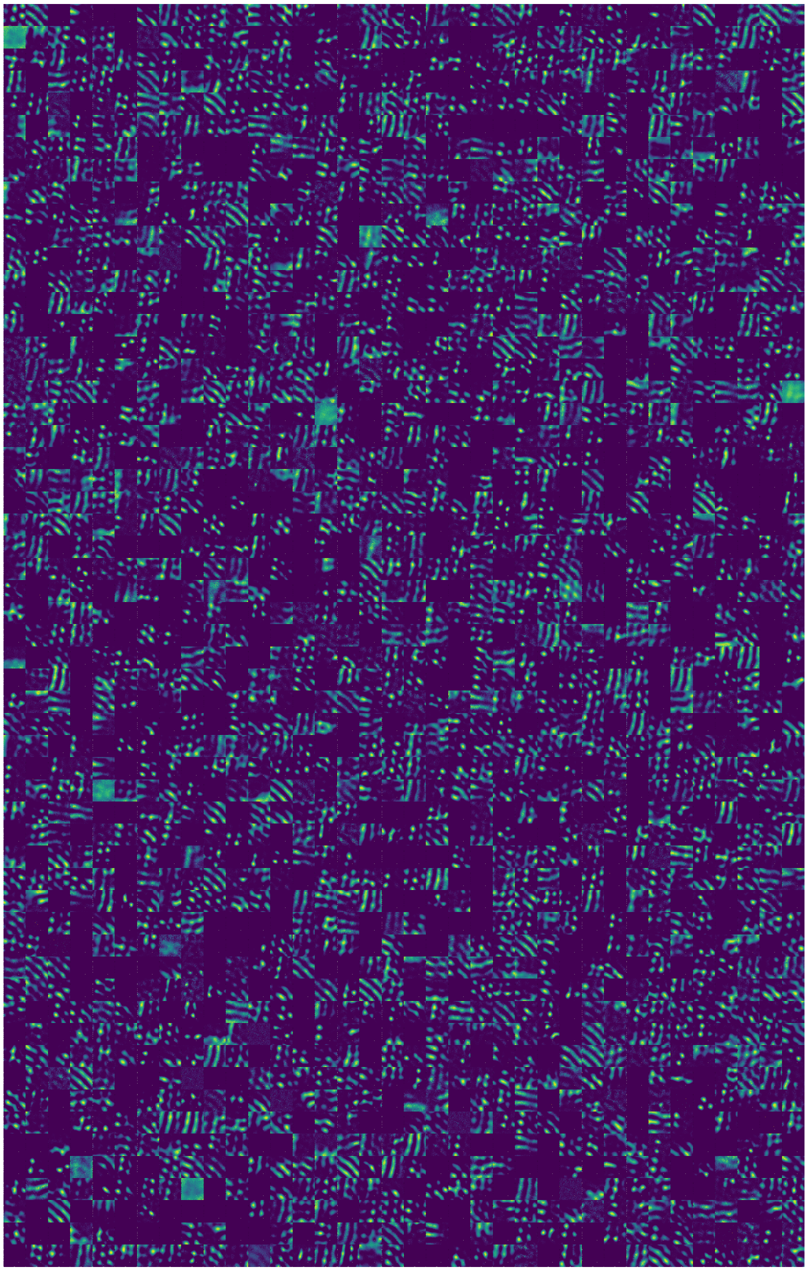

Figure S9: **2052 ratemap examples from familiar environment Environment 2, related to Fig. 2**. Each ratemap in a given position is comparable to the ratemaps from familiar environment Environment 0 (fig. S7) and Environment 1 (fig. S8) (identical cells, different environment).
